# Evaluation of Large Language Models in the Clinical Management of Patients With Upper Gastrointestinal Bleeding: Insights From Real‐World Patient Data

**DOI:** 10.1002/deo2.70373

**Published:** 2026-07-09

**Authors:** Mohsen Rajabnia, Farbod Davoodi, Mahsa Hajisafarali, Shadi Asadinia, Mahsa Shiri, Fatemeh Shirzad, Peyman Saeedi, Mahsa Mohammadi

**Affiliations:** ^1^ Department of Medicine Alborz University of Medical Sciences Karaj Iran; ^2^ Department of Computer Science Missouri University of Science and Technology Rolla Missouri USA

**Keywords:** artificial intelligence, clinical decision support, endoscopy, gastrointestinal bleeding, large language models

## Abstract

**Objective:**

To evaluate large language models (LLMs) for pre‐endoscopy (PE) risk stratification and prediction of endoscopic findings in upper gastrointestinal bleeding (UGIB), and compare their performance with clinical risk scores, conventional machine learning (ML) models, and hybrid approaches.

**Methods:**

This multicenter retrospective study included 384 patients with UGIB who underwent endoscopy. Five LLMs (GPT‐5, Gemini‐2.5‐Flash, Llama 4, Grok, and DeepSeek R1) were tested using structured zero‐shot prompts based on PE clinical and laboratory data. Their performance in identifying high‐risk patients was compared with the Glasgow‐Blatchford Score (GBS), AIMS65, PE Rockall score, and ML models. Hybrid LLM–score models were also assessed. Two gastroenterologists evaluated the quality of LLM‐generated justifications.

**Results:**

GBS showed the best discriminative performance among clinical scores (area under the receiver operating characteristic curve [AUROC] 0.73). Among LLMs, GPT‐5 achieved the highest accuracy (0.66), while Grok showed the best‐balanced performance (0.59; F1 0.47). Gemini‐2.5‐Flash had the highest sensitivity (0.89) but low specificity (0.21). Endoscopic prediction performance was modest, with Gemini‐2.5‐Flash achieving the highest exact‐match accuracy (0.34) and micro‐F1 (0.38). Hybrid models improved performance over standalone LLMs but did not outperform GBS alone (best: GBS+GPT‐5, AUROC 0.670. LLMs showed higher numerical performance than conventional ML models, but a statistical comparison was not possible due to unavailable instance‐level data. Grok received the highest human evaluation score for explanation quality.

**Conclusions:**

LLMs showed moderate performance in UGIB risk stratification and endoscopic prediction but were inferior to clinical scores, especially GBS. Hybrid models modestly improved over standalone LLMs but not GBS, supporting their use as adjunct tools rather than clinical decision‐support systems.

**Trial Registration:**

N/A

## Graphical abstract

Large language models are evaluated for pre‐endoscopy risk stratification and prediction of endoscopic findings in patients with upper gastrointestinal bleeding. Their performance is compared with validated clinical risk scores, conventional machine learning models, and hybrid approaches. Although hybrid models show modest improvements over standalone large language models, the Glasgow‐Blatchford Score remains the most accurate tool for clinical risk assessment.

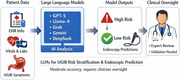

## 1 Introduction

Gastrointestinal bleeding (GIB) is a common emergency requiring rapid risk assessment and timely management. Upper GIB (UGIB) accounts for over half of cases, with 2%–10% mortality [1]. Urgent endoscopy is often performed at night or on holidays, though bleeding may have already stopped. Guidelines recommend stratifying patients into high‐risk, needing prompt interventions (transfusions or hemostatic procedures—endoscopic, surgical, or interventional radiology), and low‐risk, who can be safely discharged for outpatient care [1, 2]. Validated scores—including Glasgow‐Blatchford Score (GBS), pre‐endoscopy (PE) Rockall, and AIMS65—predict risk and mortality [3]. Effective risk stratification reduces unnecessary endoscopy and improves care efficiency; however, the predictive performance and clinical adoption of existing tools remain limited [1, 2].

Rapid advances in large language models (LLMs) have driven their widespread adoption and demonstrated capacity to comprehend and generate complex medical information [4, 5, 6]. Clinicians increasingly integrate these tools into practice. Compared with human specialists, LLMs exhibit broad understanding across medical domains, highlighting their potential as diagnostic support tools [4]. Conventional risk scores often fail to capture the complex interplay of clinical, laboratory, and endoscopic factors affecting outcomes. LLMs are simple to implement, integrate diverse data, and provide context‐specific predictions. These features make them promising tools for text classification in clinical decision support [7, 8].

However, their role in UGIB risk stratification has not been systematically evaluated, particularly in comparison with validated clinical risk scores. Therefore, this study aimed to evaluate the ability of LLMs to classify patients with UGIB as high‐risk or low‐risk using PE clinical data and to predict probable endoscopic findings. In addition, LLM performance was compared with established clinical risk scores and conventional machine learning (ML) models, and hybrid approaches combining LLMs with validated scoring systems were assessed to determine their potential additive value in acute clinical decision‐making. Additionally, the study evaluated the quality and clinical relevance of LLM‐generated justifications through independent review by two gastroenterologists to determine their potential as decision‐support tools in the acute care setting.

## 2 Methods

### 2.1 Study Design and Patient Selection

This multicenter retrospective observational study was conducted at two referral centers affiliated with Alborz University of Medical Sciences between March and December 2024. Both centers provide 24‐h endoscopic and critical care services. The study was approved by the institutional ethics committee (IR.ABZUMS.REC.1403.202), and written informed consent was obtained from all participants.

Patients presenting to the emergency department with suspected UGIB (hematemesis, coffee‐ground emesis, melena, or hematochezia of presumed upper gastrointestinal origin) were eligible if they underwent upper gastrointestinal endoscopy during hospitalization. Patients without endoscopy or with incomplete clinical records were excluded.

A standardized dataset was extracted from Electronic Health Records (EHRs), including demographics, symptoms, comorbidities, medication and lifestyle history, admission vital signs, physical examination findings, laboratory results, and endoscopic findings. Data were annotated using predefined variable definitions (Table 1) by four trained researchers. A random 20% subset was independently double‐annotated, and inter‐rater agreement was assessed using Cohen's κ. Discrepancies were resolved by consensus discussion.

**TABLE 1:** Clinical and demographic variables extracted from Electronic Health Records (EHRs) of patients presenting with upper gastrointestinal bleeding (UGIB).

Domain	Variable	Description
Demographics	Age	Recorded in years
	Sex	Male/Female
Presenting features	Melena/Hematochezia	Presence of black, tarry, or bloody stools (Yes/No)
	Hematemesis	Bloody vomiting (Yes/No)
	Syncope	Transient loss of consciousness (Yes/No)
	Shock	Hemodynamic instability at presentation (Yes/No)
Comorbidities	Diabetes mellitus	Documented diagnosis (Yes/No)
	Hypertension	Documented diagnosis (Yes/No)
	Cardiovascular disease	Ischemic heart disease, heart failure, or arrhythmia (Yes/No)
	Deep vein thrombosis	History of DVT (Yes/No)
	Chronic obstructive pulmonary disease	Documented COPD (Yes/No)
	Pulmonary embolism	History of PE (Yes/No)
	Chronic liver disease	Non‐cirrhotic liver disease (Yes/No)
	Cirrhosis	Confirmed diagnosis of cirrhosis (Yes/No)
	Chronic renal disease	Chronic kidney disease (Yes/No)
	Anemia	Pre‐existing anemia (Yes/No)
Lifestyle & medications	Smoking	Active tobacco use (Yes/No)
	Alcohol use	Documented alcohol consumption (Yes/No)
	Peptic ulcer disease	History of PUD (Yes/No)
	Previous GIB	History of UGIB or LGIB (Yes/No)
	Anti‐acid therapy	Proton pump inhibitors or H2‐blockers (Yes/No)
	NSAID use	Non‐steroidal anti‐inflammatory drugs (Yes/No)
	Anticoagulant use	Warfarin, DOACs, or heparin (Yes/No)
Clinical parameters	Mental status	Normal/Altered
	Blood pressure	Measured in mmHg
	Heart rate	Beats per minute
Laboratory values	Hemoglobin (Hb)	g/dL (at admission, 6 h, and 12 h after)
	Albumin	g/dL
	Blood urea nitrogen (BUN)	mg/dL
	Creatinine	mg/dL
	C‐reactive protein (CRP)	mg/L
	Platelet count	×10^3^/µL
	Prothrombin time (PT)	Seconds
	Partial thromboplastin time (PTT)	Seconds
	International normalized ratio (INR)	Ratio
Endoscopy	Endoscopic findings	Endoscopic findings, including lesion type and when a peptic ulcer was present, and the corresponding Forrest classification

Abbreviations: BUN, blood urea nitrogen; COPD, chronic obstructive pulmonary disease; CRP, C‐reactive protein; DOAC, direct oral anticoagulant; DVT, deep vein thrombosis; Hb, hemoglobin; INR, international normalized ratio; LGIB, lower gastrointestinal bleeding; NSAID, non‐steroidal anti‐inflammatory drug; PT, prothrombin time; PTT, partial thromboplastin time; UGIB, upper gastrointestinal bleeding. Categorical variables were recorded as binary values (Yes/No). Laboratory measurements were obtained according to institutional protocols.

### 2.2 Data Collection and Clinical Variables

A total of 536 patients were initially identified. Records with missing data were excluded during the data preprocessing stage. In total, 384 cases met eligibility criteria. All data were entered into a structured Excel spreadsheet and checked for accuracy and completeness. PE data were used for prediction, and Endoscopic findings were not included in model inputs and were used as outcome labels. Collected variables are summarized in Table 1.

### 2.3 Outcome Definition and Risk Stratification

Patients were stratified into high‐ and low‐risk groups based on PE clinical and laboratory features. High‐risk patients were defined as those with hemodynamic instability (systolic blood pressure <90 mmHg, heart rate >100 beats/min, syncope, or shock), evidence of ongoing bleeding, or suspected variceal hemorrhage, requiring urgent endoscopic intervention. Low‐risk patients were those without these features and were considered suitable for outpatient management.

In addition, the LLMs were tasked with predicting endoscopic findings, which included peptic ulcer disease (Forrest I–III), erosive gastropathy, esophageal varices, Mallory–Weiss tears, tumors, and other gastrointestinal lesions.

### 2.4 Clinical Risk Score Comparison

To contextualize the performance of LLMs within established clinical practice, validated PE risk stratification tools were additionally evaluated on the same cohort of 384 patients. The GBS, AIMS65 score, and PE Rockall score were calculated for each patient based on established guideline criteria using data extracted from EHRs.

For each scoring system, multiple decision thresholds were assessed to evaluate performance across different sensitivity–specificity tradeoffs. Patients were classified as high‐risk or low‐risk according to each threshold, and predictive performance was calculated against the reference clinical outcome. Evaluation metrics included accuracy, sensitivity, specificity, positive predictive value (PPV), negative predictive value (NPV), F1‐score, and area under the receiver operating characteristic curve (AUROC).

### 2.5 Conventional ML Models Evaluation

Conventional ML models were generated using the LazyPredict framework to provide benchmark comparison across standard classification algorithms. The dataset was divided into independent training (80%) and testing (20%) sets using stratified sampling with a fixed random seed to preserve class distribution and prevent data leakage. Model performance was evaluated on the held‐out test set using accuracy, F1‐score, and computation time (wall‐clock seconds), with additional stratified 5‐fold cross‐validation across the 384 patient records.

### 2.6 LLMs Evaluation

LLM evaluation used structured prompts incorporating each patient's PE clinical and laboratory data. Models were assessed in a zero‐shot setting with internal chain‐of‐thought [11, 12] and tree‐of‐thought [13] reasoning to support stepwise clinical inference; however, only final structured outputs were used for quantitative evaluation. Prompts utilized a zero‐shot approach to prevent dataset overlap and performance inflation. Each query detailed clinical symptoms to mandate a risk‐intervention assessment. Outputs were strictly constrained to a Tree‐of‐Thought format containing three predefined components. Every prompt was executed independently across all LLMs, with outputs evaluated via standardized baseline metrics. Preliminary models (MedGemma [14], MedAlpaca [15], Meditron [16], and BERT variants [17, 18, 19]) were excluded due to narrow context windows, infrastructure constraints, or insufficient reasoning capacity. Final benchmarks prioritized models supporting extended context limits and high‐quality generation: GPT‐5 [20], Gemini‐2.5‐Flash [21], Llama 4 [22], DeepSeek R1 [23, 24], and Grok [25] (see Doc S1). Binary risk stratification was evaluated using accuracy, sensitivity, specificity, PPV, NPV, F1‐score, Matthew's correlation coefficient (MCC), Cohen's kappa, and AUROC. Multi‐label prediction of endoscopic findings was assessed using exact match accuracy, Jaccard similarity, and macro‐, micro‐, and weighted‐average F1‐scores to assess agreement between predicted and reference endoscopic diagnoses. Full prompts for binary and multi‐label tasks are provided in Doc S3.

### 2.7 Hybrid Model Development

To evaluate whether integration of traditional clinical risk scores with LLM predictions could improve predictive performance, hybrid models were developed by combining binary predictions from LLMs with established clinical scoring systems, including GBS, AIMS65, and PE Rockall scores.

Hybrid models were evaluated on the same cohort using accuracy, sensitivity, specificity, PPV, NPV, F1‐score, and AUROC. This framework enabled assessment of whether LLM‐derived reasoning provided additive value beyond validated clinical risk stratification tools alone.

### 2.8 Human Evaluation of LLM Justifications

Two board‐certified gastroenterologists independently reviewed a random subset of 100 cases to evaluate the quality and clinical reliability of LLM‐generated justifications from Llama 4, GPT‐5, Gemini‐2.5‐Flash, Grok, and DeepSeek R1.

Outputs were assessed across six domains:

1. Relevance—alignment with the patient's health record.
2. Clarity—readability and comprehensibility.
3. Completeness—coverage of all relevant aspects of the record.
4. Specificity—precision in addressing the prediction.
5. Correctness—factual accuracy and consistency with clinical data.
6. Consistency—logical coherence and alignment with documented reasoning.

Each domain was scored on a 3‐point Likert scale (1 = poor, 3 = excellent). Although a structured framework was used, the non‐blinded assessment may have introduced subjective bias.

### 2.9 Statistical Analysis

All statistical analyses were performed using Python‐based scientific computing libraries. Ninety‐five percent confidence intervals for accuracy estimates were calculated using the Wilson score method. Conventional ML models were run in Google Colab on an NVIDIA T4 GPU, while LLM inference was performed via API on an NVIDIA A100 (40 GB) GPU (Google Colab Pro). Due to the lack of instance‐level outputs from LazyPredict, paired tests such as McNemar's test were not applicable.

## 3 Results

### 3.1 Dataset Characteristics

The cohort included 384 patients (≈106 high‐risk, 278 low‐risk), reflecting class imbalance. All models (clinical scores, ML models, and LLMs) were evaluated on the same dataset with an identical class distribution. Inter‐rater agreement among clinicians was substantial to near‐perfect (Cohen's κ = 0.89), indicating high annotation consistency.

### 3.2 Clinical Risk Score Comparison

Table 2 shows the diagnostic performance of three risk scores—GBS, AIMS65, and PE Rockall score—across different cutoff thresholds for predicting high‐risk UGIB patients.

**TABLE 2:** Performance of the Glasgow‐Blatchford Score (GBS), AIMS65, and pre‐endoscopy (PE) Rockall scores for prediction of high‐risk upper gastrointestinal bleeding (UGIB) across different thresholds.

Score	Threshold	Accuracy	Sensitivity	Specificity	PPV	NPV	F1	AUROC
**GBS**	1	0.624021	0.96087	0.117647	0.620787	0.666667	0.754266	0.728815
	2	0.663185	0.934783	0.254902	0.653495	0.722222	0.769231	0.728815
	3	0.684073	0.886957	0.379085	0.682274	0.690476	0.771267	0.728815
	4	0.694517	0.834783	0.48366	0.708487	0.660714	0.766467	0.728815
	5	0.699739	0.795652	0.555556	0.729084	0.643939	0.760915	0.728815
	6	0.684073	0.734783	0.607843	0.737991	0.603896	0.736383	0.728815
	7	0.678851	0.678261	0.679739	0.760976	0.58427	0.717241	0.728815
	8	0.668407	0.626087	0.732026	0.778378	0.565657	0.693976	0.728815
	9	0.631854	0.534783	0.777778	0.783439	0.526549	0.635659	0.728815
	10	0.603133	0.456522	0.823529	0.795455	0.501992	0.58011	0.728815
	11	0.571802	0.378261	0.862745	0.805556	0.48	0.514793	0.728815
	12	0.535248	0.278261	0.921569	0.842105	0.459283	0.418301	0.728815
	13	0.506527	0.221739	0.934641	0.836066	0.444099	0.350515	0.728815
	14	0.462141	0.121739	0.973856	0.875	0.424501	0.21374	0.728815
	15	0.428198	0.052174	0.993464	0.923077	0.410811	0.098765	0.728815
	16	0.409922	0.021739	0.993464	0.833333	0.403183	0.042373	0.728815
	17	0.4047	0.008696	1	1	0.401575	0.017241	0.728815
**AIMS65**	1	0.543081	0.456522	0.673203	0.677419	0.451754	0.545455	0.565473
	2	0.43342	0.104348	0.928105	0.685714	0.408046	0.181132	0.565473
	3	0.4047	0.017391	0.986928	0.666667	0.400531	0.033898	0.565473
	4	0.402089	0.004348	1	1	0.400524	0.008658	0.565473
**pre‐endoscopy Rockall**	1	0.56658	0.665217	0.418301	0.632231	0.453901	0.648305	0.577621
	2	0.543081	0.465217	0.660131	0.672956	0.450893	0.550129	0.577621
	3	0.51436	0.334783	0.784314	0.7	0.43956	0.452941	0.577621
	4	0.483029	0.191304	0.921569	0.785714	0.431193	0.307692	0.577621
	5	0.438642	0.086957	0.96732	0.8	0.413408	0.156863	0.577621
	6	0.399478	0.017391	0.973856	0.5	0.397333	0.033613	0.577621
	7	0.396867	0	0.993464	0	0.397906	0	0.577621

Performance metrics of the GBS, AIMS65, and PE Rockall score are reported across multiple decision thresholds to illustrate the sensitivity–specificity trade‐off. All models were evaluated on the same cohort of 384 patients (106 high‐risk and 278 low‐risk cases) using a unified reference standard for high‐risk UGIB classification. At each threshold, patients were classified as high‐risk or low‐risk and compared against the reference outcome. AUROC reflects overall discriminative ability, and additional metrics include accuracy, sensitivity, specificity, PPV, NPV, and F1‐score.

GBS demonstrated the best discriminative ability, with the highest AUROC (0.7288), compared with AIMS65 (0.5655) and PE Rockall (0.5776). This indicates that GBS had superior overall performance in distinguishing high‐risk from low‐risk patients.

Taken together, these findings indicate that GBS outperformed both AIMS65 and PE Rockall score in predicting high‐risk UGIB patients, particularly at intermediate thresholds where sensitivity and specificity were more balanced. A detailed analysis of threshold‐dependent diagnostic performance for all risk scores is presented in Doc S2.

### 3.3 Application of Conventional ML Models

We assessed conventional ML models, and Accuracy, F1‐score, and computation time for each model are presented in Table 3. Computation time was reported as the mean inference time (seconds) under identical hardware conditions. Compared with established clinical risk scores, conventional ML models demonstrated only moderate predictive performance. Among the evaluated models, LabelSpreading achieved the highest F1‐score (0.52), while BaggingClassifier provided balanced accuracy with minimal computation time. However, overall performance remained inferior to GBS, which demonstrated superior numerical results for high‐risk UGIB prediction. As paired statistical testing could not be performed, no inference regarding statistical superiority can be made.

**TABLE 3:** Comparative performance metrics of conventional machine learning (ML) models.

Model	Accuracy	F1‐Score	Computation time
Bagging Classifier	0.49	0.48	0.07
Label Spreading	0.54	0.52	0.11
K Neighbors Classifier	0.49	0.46	0.05
Extra Tree Classifier	0.44	0.44	0.02
Ada Boost Classifier	0.32	0.21	0.18
Decision Tree Classifier	0.39	0.38	0.04
LGBM Classifier	0.37	0.36	0.32
Bernoulli NB	0.37	0.33	0.03
Random ForestClassifier	0.37	0.34	0.41
SVC	0.29	0.25	0.09
Ridge Classifier CV	0.29	0.26	0.06
Ridge Classifier	0.29	0.26	0.02
Logistic Regression	0.29	0.26	0.04
Linear DiscriminantAnalysis	0.29	0.26	0.05
Linear SVC	0.29	0.26	0.07
Dummy Classifier	0.24	0.10	0.04
Calibrated Classifier CV	0.24	0.13	0.18
Passive AggressiveClassifier	0.24	0.23	0.04
Nearest Centroid	0.12	0.14	0.05
SGD Classifier	0.20	0.18	0.13
Perceptron	0.20	0.17	0.02
Gaussian NB	0.12	0.17	0.02

Performance metrics were computed on the same held‐out test dataset for all models using a consistent evaluation pipeline. Accuracy and F1‐score are reported as unitless proportions. Computation time represents the mean wall‐clock inference time (seconds) measured under identical hardware conditions. Conventional machine learning models were evaluated on the same test dataset using the experimental framework described in the Methods section.

### 3.4 Application of LLMs

Performance of LLMs in predicting high‐ versus low‐risk UGIB patients (Table 4) was overall moderate with marked inter‐model variability. GPT‐5 achieved the highest accuracy (0.66; 95% confidence interval: 0.61–0.71), followed by Llama 4 (0.64; 0.59–0.69), DeepSeek R1 (0.57; 0.52–0.62), Grok (0.54; 0.49–0.59), and Gemini‐2.5‐Flash (0.40; 0.35–0.45). Balanced accuracy remained low across models (0.54–0.59), with weak agreement metrics (κ: 0.06–0.13; MCC: 0.07–0.16), indicating limited discrimination beyond chance and effects of class imbalance.

**TABLE 4:** Performance of large language models (LLMs) in classifying patients into high‐ and low‐risk groups and multi‐label prediction of endoscopic findings.

Metric	Llama 4	Gemini‐2.5‐Flash	GPT‐5	Grok	DeepSeek R1
**Overall performance**					
Accuracy	0.64	0.40	0.66	0.54	0.57
Balanced accuracy	0.55	0.55	0.54	0.59	0.54
Cohen's κ	0.10	0.06	0.09	0.13	0.06
Matthew's correlation coefficient	0.10	0.11	0.09	0.16	0.07
Precision	0.36	0.31	0.37	0.35	0.33
Recall (Sensitivity)	0.35	0.89	0.26	0.70	0.46
F1‐Score	0.35	0.46	0.31	0.47	0.38
Specificity	0.75	0.21	0.82	0.47	0.61
**Multi‐label endoscopy results**					
Exact match accuracy	0.20	0.34	0.29	0.32	0.21
Jaccard Score	0.20	0.33	0.29	0.31	0.21
F1‐Score (Macro)	0.02	0.30	0.27	0.27	0.22
F1‐Score (Micro)	0.23	0.38	0.30	0.36	0.25
F1‐Score (Weighted)	0.23	0.30	0.30	0.28	0.22
Computational time (s)	11.5	5	29.6	17.3	83.5

Abbreviations: FNR, false negative rate; FPR, false positive rate; LLMs, large language models; NPV, negative predictive value; TNR, true negative rate; TPR, true positive rate. Overall performance metrics reflect binary classification of patients into high‐risk and low‐risk groups using PE clinical and laboratory data. Macro‐averaged metrics represent unweighted means across classes, micro‐averaged metrics reflect global performance across all samples, and weighted metrics account for class imbalance. Multi‐label endoscopy results evaluate the prediction of endoscopic findings, with exact match accuracy indicating complete label agreement, partial match accuracy, and Jaccard score reflecting partial overlap, and F1‐scores reported using macro, micro, and weighted averaging. Computational time (s) represents the mean wall‐clock inference time (seconds) per model under identical hardware conditions. Computational runtime measurements were obtained utilizing an NVIDIA A100 Tensor Core GPU infrastructure on Google Colab Pro for LLM evaluation workloads.

**FIGURE 1:**
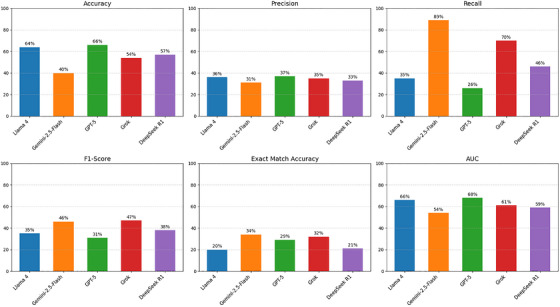
Performance of evaluated large language models across key metrics, including accuracy, precision, recall, F1‐score, exact match accuracy, and area under the curve (AUC).

Grok showed the most balanced performance, with the highest balanced accuracy (0.59), MCC (0.16), κ (0.13), and F1‐score (0.47). Gemini‐2.5‐Flash prioritized sensitivity (0.89) but had low specificity (0.21) and a high false‐positive rate (0.79), while GPT‐5 showed the opposite pattern with the highest specificity (0.82) but low sensitivity (0.26) and the highest false‐negative rate (0.74). Llama 4 and DeepSeek R1 showed intermediate performance with moderate specificity and NPV but persistently low sensitivity (Figure 1).

Class‐wise, high‐risk F1‐scores ranged from 0.31 (GPT‐5) to 0.47 (Grok), and low‐risk F1‐scores from 0.34 (Gemini‐2.5‐Flash) to 0.78 (GPT‐5), reflecting divergent precision–recall trade‐offs and limited reliability for autonomous triage (Table S1). AUROC analysis confirmed modest discrimination overall, with Grok demonstrating the most balanced performance despite GPT‐5's higher specificity and Gemini‐2.5‐Flash's sensitivity‐driven profile.

For multi‐label endoscopic prediction, Gemini‐2.5‐Flash performed best (exact‐match 0.34, Jaccard 0.33, micro‐F1 0.38), followed by Grok and GPT‐5, though overall performance remained low (exact‐match 0.20–0.34; macro‐F1 0.02–0.30).

Compared with clinical risk scores, LLMs were inferior in discrimination, with GBS achieving the highest AUROC (0.73) and stronger sensitivity.

Computation time varied widely: Gemini‐2.5‐Flash was fastest (5 s), DeepSeek R1 slowest (83.5 s), GPT‐5 intermediate‐high (29.6 s), with Llama 4 and Grok in between.

### 3.5 Application of Hybrid Models

Hybrid models combining validated clinical risk scores with LLM predictions were evaluated for complementary value in clinical risk stratification (Table 5). Overall, hybrid approaches outperformed standalone LLMs, particularly when integrated with GBS, but did not outperform GBS alone.

**TABLE 5:** Diagnostic performance of hybrid models combining clinical risk scores with large language models (LLMs) for the prediction of high‐risk upper gastrointestinal bleeding (UGIB) patients.

Model type	Model	Accuracy	Sensitivity	Specificity	PPV	NPV	F1‐score	AUROC
Llama 4	GBS plus Llama 4	0.674	0.838	0.434	0.684	0.647	0.753	0.636
	PE Rockall plus Llama 4	0.634	0.752	0.461	0.671	0.560	0.709	0.606
	AIMS65 plus Llama 4	0.626	0.757	0.434	0.661	0.550	0.706	0.595
DeepSeek R1	GBS plus DeepSeek R1	0.648	0.770	0.464	0.686	0.569	0.725	0.617
	AIMS65 plus DeepSeek R1	0.583	0.670	0.450	0.650	0.472	0.660	0.560
	PE Rockall plus DeepSeek R1	0.559	0.635	0.444	0.635	0.444	0.635	0.539
Grok	GBS plus Grok	0.678	0.747	0.575	0.725	0.603	0.735	0.661
	AIMS65 plus Grok	0.636	0.686	0.562	0.701	0.544	0.693	0.624
	PE Rockall plus Grok	0.634	0.694	0.542	0.694	0.542	0.694	0.618
Gemini‐2.5‐Flash	GBS plus Gemini‐2.5‐Flash	0.616	0.896	0.205	0.623	0.574	0.735	0.551
	AIMS65 plus Gemini‐2.5‐Flash	0.608	0.891	0.192	0.618	0.547	0.730	0.542
	PE Rockall plus Gemini‐2.5‐Flash	0.608	0.891	0.192	0.618	0.547	0.730	0.542
GPT‐5	GBS plus GPT‐5	0.685	0.736	0.604	0.746	0.592	0.741	0.670
	AIMS65 plus GPT‐5	0.585	0.586	0.583	0.689	0.472	0.633	0.585
	PE Rockall plus GPT‐5	0.558	0.511	0.632	0.686	0.450	0.586	0.571

Abbreviations: AUROC, area under the receiver operating characteristic curve; GBS, Glasgow‐Blatchford Score; NPV, negative predictive value; Rockall_pre, PE Rockall score; PPV, positive predictive value. Hybrid models were generated by combining conventional clinical risk scores with outputs from LLMs. Diagnostic performance metrics are reported for the prediction of high‐risk UGIB patients in the test dataset. Higher AUROC values indicate better discriminative performance.

GBS + GPT showed the best performance among all hybrids, with the highest accuracy (0.685), specificity (0.604), PPV (0.746), F1‐score (0.741), and AUROC (0.670), while maintaining a balanced sensitivity–specificity profile. GBS + Grok also performed strongly (accuracy 0.678, AUROC 0.661). In contrast, GBS + Gemini achieved the highest sensitivity (0.896) but low specificity (0.205), indicating over‐classification of high‐risk patients. GBS + Llama showed high sensitivity (0.838) with moderate discrimination (AUROC 0.636).

Hybrids using AIMS65 or PE Rockall scores performed worse than GBS‐based combinations. Across nearly all LLMs, GBS integration consistently yielded the strongest results, suggesting improved predictive discrimination when combining clinical risk scores with LLM reasoning.

However, overall AUROC values remained modest (0.539–0.670), indicating limited standalone utility for high‐stakes decisions. Hybrid models exhibited architecture‐dependent trade‐offs: Gemini‐based models favored sensitivity, while GPT‐ and Grok‐based models achieved more balanced performance.

### 3.6 Human Justification Evaluation Results

Among the evaluated models’ justifications, Grok achieved the highest overall human evaluation score (2.63), followed by Gemini 2.5 Flash (2.40), DeepSeek R1 (2.28), GPT‐5 (2.13), and Llama 4 (1.68) (Table 6). Grok consistently scored highest across quality domains, particularly clarity (2.99 ± 0.11) and relevance (2.97 ± 0.16), whereas Llama 4 performed worst, with notably low completeness (1.02 ± 0.14) and clarity (1.82 ± 0.87). GPT‐5 showed moderate performance but lower completeness and specificity than Grok and Gemini.

**TABLE 6:** Human evaluation of large language model (LLM) outputs across quality dimensions.

Model	Relevance (Mean ± SD)	Clarity (Mean ± SD)	Completeness (Mean ± SD)	Specificity (Mean ± SD)	Correctness (Mean ± SD)	Consistency (Mean ± SD)	Overall Score
Gemini2.5 Flash	2.76 ± 0.61	2.75 ± 0.50	2.39 ± 0.85	2.34 ± 0.75	1.92 ± 0.94	2.24 ± 0.86	2.40
DeepSeek R1	2.67 ± 0.59	2.66 ± 0.56	2.01 ± 0.93	2.31 ± 0.75	1.80 ± 0.94	2.15 ± 0.91	2.28
Grok	2.97 ± 0.16	2.99 ± 0.11	2.35 ± 0.73	2.53 ± 0.64	2.44 ± 0.83	2.51 ± 0.80	2.63
GPT‐5	2.32 ± 0.86	2.42 ± 0.88	1.67 ± 0.53	1.84 ± 0.71	2.32 ± 0.86	2.25 ± 0.85	2.13
LLaMA4	1.91 ± 0.78	1.82 ± 0.87	1.02 ± 0.14	1.53 ± 3.28	2.17 ± 0.89	1.63 ± 0.74	1.68

Values are presented as mean ± standard deviation. Scores were based on human evaluation using a Likert‐type scale. The overall score represents the average of all evaluated dimensions.

Qualitative assessment revealed distinct model behaviors. GPT‐5 generated generally complete but sometimes repetitive justifications and tended to over‐classify urgent cases when GIB co‐occurred with tachycardia. Llama 4 produced brief, vague, and low‐specificity explanations with limited reasoning. Grok provided the most comprehensive justifications, although reasoning was occasionally inconsistent. Gemini 2.5 Flash incorporated multiple clinical features into structured explanations but frequently misclassified cases because of incorrect feature prioritization. DeepSeek R1 generated detailed patient summaries and structured predictions, but its explanations often did not clearly justify the predictions and occasionally favored overtreatment. Overall, substantial variation was observed among models in justification depth, reasoning consistency, and predictive accuracy.

## 4 Discussion

In this multicenter retrospective study, we evaluated contemporary LLMs for PE UGIB risk stratification and prediction of endoscopic findings against validated clinical risk scores and conventional ML models. Overall, validated scores, particularly GBS, outperformed standalone LLMs and ML models, while hybrid models combining LLMs with established scores yielded modest improvements.

GBS demonstrated the best discriminative performance, achieving the highest AUROC and a more balanced sensitivity–specificity profile than AIMS65, PE Rockall score, ML models, and LLMs. Conventional ML models demonstrated only moderate performance and showed lower numerical results compared with GBS and LLMs; however, because paired statistical testing could not be performed, no conclusions regarding statistical differences can be drawn.

LLMs exhibited heterogeneous behavior. GPT‐5 achieved the highest accuracy and specificity but low sensitivity, potentially missing high‐risk patients. Gemini‐2.5‐Flash showed the highest sensitivity but poor specificity, suggesting over‐classification of high‐risk cases. Grok demonstrated the most balanced performance, achieving the highest balanced accuracy, MCC, Cohen's κ, and F1‐score, although overall agreement‐based metrics remained modest, reflecting limited discrimination and class imbalance. These findings indicate that current LLMs are not sufficiently reliable for autonomous UGIB triage.

Hybrid models, particularly GBS + GPT‐5 and GBS + Grok, improved discrimination compared with standalone LLMs, but did not outperform GBS alone, supporting an adjunctive rather than replacement role for established risk scores.

Prediction of endoscopic findings remained challenging, although Gemini‐2.5‐Flash achieved the highest exact‐match accuracy and micro‐F1 score. Human evaluation revealed marked differences in justification quality: Grok received the highest qualitative ratings, whereas Llama 4 demonstrated the weakest reasoning. Notably, explanation quality did not consistently correlate with predictive performance.

Strengths of this study include direct head‐to‐head comparison of multiple LLMs, validated risk scores, and ML models within the same cohort; evaluation of hybrid approaches; and structured gastroenterologist assessment of model‐generated reasoning. High inter‐rater agreement (κ = 0.89) supported annotation reliability.

Compared with previous studies reporting strong LLM performance in gastroenterology, our findings were more conservative. Prior work reported AUCs up to 0.986 for GI bleeding detection using nursing notes [7], superior diagnostic coverage by Claude 3.5 Sonnet in complex GI cases [4, 9], and improved performance of the domain‐specific GutGPT model trained on >190,000 GI question–answer pairs [10]. The lower performance observed in our study likely reflects the greater complexity of real‐world retrospective EHR data, zero‐shot prompting, and the absence of domain‐specific fine‐tuning.

Limitations include the retrospective design, potential documentation and selection bias, inclusion only of patients undergoing endoscopy, and conduct at two referral centers within a single geographic region. Human justification assessment remained partially subjective and non‐blinded, with a limited sample size. Furthermore, all LLMs were evaluated using zero‐shot prompting, and prediction‐level outputs from ML models were unavailable for paired statistical comparisons.

In conclusion, contemporary LLMs demonstrated moderate performance for UGIB risk stratification and prediction of endoscopic findings but remained inferior to validated clinical risk scores, particularly GBS. Hybrid approaches provided modest gains, suggesting potential value as adjunctive clinical decision‐support tools rather than standalone systems for high‐stakes UGIB triage.

## Author Contributions

**Mohsen Rajabnia** designed the study, performed human model evaluation, and supervised the project. **Farbod Davoodi** developed the model and drafted the manuscript. **Mahsa Hajisafarali** designed the study, collected the clinical data, performed data preprocessing, and drafted the manuscript. **Shadi Asadinia** collected the clinical data and edited the manuscript. **Mahsa Shiri** collected the clinical data and edited the manuscript. **Fatemeh Shirzad** collected the clinical data and edited the manuscript. **Peyman Saeedi** collected the clinical data and edited the manuscript. **Mahsa Mohammadi** performed human model evaluation, edited the manuscript, and supervised the project. All authors read and approved the final manuscript.

## Funding

The authors have nothing to report.

## Artificial Intelligence Use Disclosure

ChatGPT (OpenAI and GPT‐5) was used solely for language editing and improvement of manuscript clarity after completion of all data analyses and benchmarking procedures (grammar, clarity, and style). The artificial intelligence (AI) tool was not used for study design, data processing, statistical analysis, or interpretation of results. All scientific analyses, results, and conclusions were generated independently by the authors. All AI‐assisted edits were reviewed and approved by the authors. The final manuscript reflects the authors’ interpretation and conclusions.

## Ethics Statement

This study was approved by the Institutional Ethics Committee of Alborz University of Medical Sciences (IR.ABZUMS.REC.1403.202). All patient data were fully anonymized before analysis, and the study was conducted in accordance with the ethical principles of the Declaration of Helsinki.

## Consent

Written informed consent was obtained from all patients.

## Conflicts of Interest

The authors declare no conflicts of interest.

## Supporting information




**Supporting File 1**: deo270373‐Sup‐0001‐SuppMat.docx

## Data Availability

The datasets analyzed during the current study are available from the corresponding author upon reasonable request.
