# Supplementary material for: Evaluation of Large Language Models in the Clinical Management of Patients With Upper Gastrointestinal Bleeding: Insights From Real‐World Patient Data
Source: DEN Open. 2026 Jul 9;7(1):e70373. doi: 10.1002/deo2.70373 (PMC13347626; doi:10.1002/deo2.70373)
Supplement: Supplementary file 1 — Supporting File 1: deo270373‐Sup‐0001‐SuppMat.docx [file DEO2-7-e70373-s001.docx]

**Doc. S1: Selection of Evaluated General and Medical LLMs**

We initially evaluated several LLMs, including MedGemma[14], MedAlpaca[15], Meditron[16], MedicalBERT[17], ClinicalBERT[18], and BioBERT[19], before selecting GPT-5[20], Gemini-2.5-Flash[21], Llama 4[22], DeepSeek R1[23] [24], and Grok[25]. We excluded the former set for several reasons. First, models such as MedAlpaca, Meditron, and BERT-based variants have limited context windows, which are insufficient to process our full clinical prompts. Second, because these models have fewer parameters and narrower domain-specific fine-tuning, they often fail to achieve adequate performance on metrics such as accuracy, precision, recall, and F1-score when applied to complex medical reasoning tasks. Third, some specialized medical models require computational resources that exceed the practical limits of our infrastructure. The selected models supported larger input contexts, produced higher-quality outputs, and were compatible with our computational resources.

**Doc S2: Performance Characteristics Across Risk Score Cutoffs**

For GBS, lower thresholds (1–3) produced very high sensitivity (0.89–0.96), meaning the score identified most high-risk patients, but specificity was poor (0.12–0.38), resulting in many false positives. As the threshold increased, sensitivity gradually decreased while specificity improved. The best balance between sensitivity and specificity appeared around thresholds 4–7, where F1-score remained highest (approximately 0.72–0.77) and accuracy peaked near threshold 5 (0.70). At very high thresholds (≥12), specificity became excellent (>0.92), but sensitivity dropped markedly, meaning many true high-risk patients would be missed.

AIMS65 showed substantially lower performance overall. Even at its optimal threshold (1), sensitivity and specificity were only moderate (0.46 and 0.67, respectively), with an AUROC of 0.565. Increasing the threshold sharply reduced sensitivity while increasing specificity, indicating poor balance and limited clinical utility for screening high-risk patients.

The pre-endoscopic Rockall score performed slightly better than AIMS65 but remained inferior to GBS. At threshold 1, sensitivity was relatively high (0.67), but specificity was low (0.42). Higher thresholds improved specificity at the expense of substantial reductions in sensitivity. Its AUROC of 0.578 suggests limited discriminative performance overall.

**Table S1. Binary classification performance of LLMs in predicting high-risk and low-risk UGIB patients. Class-specific precision, recall, F1-score, and specificity are reported for Llama 4, Gemini-2.5-Flash, GPT-5, Grok, and DeepSeek R1.**

| **Metric** | **Models** | | | | | | | | | |
| --- | --- | --- | --- | --- | --- | --- | --- | --- | --- | --- |
|  | **Llama 4** | | **Gemini-2.5-Flash** | | **GPT-5** | | **Grok** | | **DeepSeek R1** | |
|  | **High risk** | **Low risk** | **High risk** | **Low risk** | **High risk** | **Low risk** | **High risk** | **Low risk** | **High risk** | **Low risk** |
| Precision | 0.36 | 0.74 | 0.31 | 0.83 | 0.37 | 0.73 | 0.35 | 0.80 | 0.33 | 0.73 |
| Recall | 0.35 | 0.75 | 0.89 | 0.21 | 0.26 | 0.82 | 0.70 | 0.47 | 0.46 | 0.61 |
| F1-Score | 0.35 | 0.75 | 0.46 | 0.34 | 0.31 | 0.78 | 0.47 | 0.59 | 0.38 | 0.66 |
| Specificity | 0.75 | 0.35 | 0.21 | 0.89 | 0.82 | 0.26 | 0.47 | 0.70 | 0.61 | 0.46 |

**Footnotes:**
Abbreviations: LLMs, large language models; High-risk patients were defined as those requiring urgent endoscopic intervention based on hemodynamic instability, evidence of ongoing bleeding, or suspected variceal hemorrhage. Low-risk patients were defined as those eligible for conservative or outpatient management. Precision, recall, F1-score, and specificity were calculated separately for high-risk and low-risk classes. Support indicates the number of true cases in each class. All metrics were calculated using PE clinical and laboratory data.

**Doc. S3.** **Structured prompt used for LLM-based UGIB risk stratification and prediction of endoscopic findings. Full prompt template including Tree-of-Thought reasoning framework, risk classification, endoscopic finding prediction, Forrest classification assessment, and standardized JSON output structure.**

“” You are an expert in the field of Gastroenterology. Your task is to analyze patient health records and predict whether urgent endoscopy is indicated, the type of endoscopy finding, and relevant classifications based on a hierarchical decision process. You will employ a Tree of Thought approach to explore decision paths before concluding.

Here is the patient data for prediction:

We have a {row['age']}-year-old {row['sex']} patient with upper gastrointestinal bleeding and the following features with this ID number {row['patient ID']}:

- Melena: {row['melena']}

- Hematemesis: {row['hematemesis']}

- Diabetes: {row['diabetes']}

- Hypertension: {row['htn']}

- Cardiovascular Disease: {row['cvd']}

- Deep Vein Thrombosis: {row['dvt']}

- COPD: {row['copd']}

- Pulmonary Embolism: {row['pulmonary_embolism']}

- Chronic Liver Disease: {row['chronic_liver_disease']}

- Cirrhosis: {row['cirrhosis']}

- Chronic Renal Disease: {row['chronic_renal_disease']}

- Anemia: {row['anemia']}

- Medications: Anti-acid: {row['anti_acid']}, NSAIDs: {row['NSAIDs']}, Anticoagulants: {row['anticoagulant']}

- Smoker: {row['smoker']}, Alcohol use: {row['alcohol']}

- History of PUD: {row['pud']}, Past GIB: {row['past_GIB']}

- Mental Status: {row['mental_status']}

- Vitals: BP: {row['bp']}, HR: {row['HR']}, Shock: {row['shock']}, Syncope: {row['syncope']}

- Labs: Hemoglobin: {row['HB(1,6,12)']}, Albumin: {row['alb']}, BUN: {row['bun']}, Creatinine: {row['cr']}, CRP: {row['crp']}, Platelets: {row['plt(*1000)']}, PT: {row['pt']}, PTT: {row['ptt']}, INR: {row['INR']}

Consider 'm' for male, 'f' for female, 'y' for yes, and 'n' for no in the input data.

---

Tree of Thought Process for each patient:

For each decision point in the endoscopy pathway, consider alternative paths or possibilities before selecting the most likely one. Provide a brief justification for why certain paths were chosen or discarded.

1. Endoscopy Indication Decision:

* Consideration 1 (e.g., Endoscopy Needed: Yes): [Reasons for needing endoscopy based on symptoms, vitals, labs, etc.]

* Consideration 2 (e.g., Endoscopy Not Needed: No): [Reasons for not needing endoscopy, if applicable (e.g., very stable, minor symptoms).]

* Final Decision for Endoscopy Needed: [Yes/No] - [Justification for the chosen decision based on the above considerations.]

2. Risk Level Decision (If Endoscopy Needed is Yes):

* Consideration 1 (e.g., Risk Level: High Risk): [Features supporting High Risk (e.g., signs of active bleeding, severe comorbidities, unstable vitals).]

* Consideration 2 (e.g., Risk Level: Low Risk): [Features supporting Low Risk (e.g., stable vitals, melena only, no significant comorbidities).]

* Final Decision for Risk Level: [High Risk/Low Risk] - [Justification for the chosen risk level.]

3. Endoscopic Finding Decision (If Risk Level determined):

* If High Risk:

* Consideration 1 (e.g., Condition: peptic Ulcer): [Features suggesting peptic Ulcer (e.g., history of PUD, NSAID use).]

* Consideration 2 (e.g., Condition: esophageal Varices): [Features suggesting esophageal varices (e.g., chronic liver disease, cirrhosis).]

* Consideration 3 (e.g., Condition: Tumor / Other Lesions): [Features suggesting malignancies, vascular lesions, etc.]

* Final Decision for High Risk Condition: [peptic Ulcer/Varice/Tumor/Other] - [Justification.]

* If Low Risk:

* Consideration 1 (e.g., Condition: Peptic Ulcer): [Features suggesting Forrest classification type 3 (F3)(e.g., clean base ulcer).]

* Consideration 2 (e.g., Condition: Erosive): [Features suggesting Erosive Gastropathy (e.g., anti-acid use, mild symptoms).]

* Consideration 3 (e.g., Condition: Mallory-Weiss): [Features suggesting Mallory-Weiss tears (e.g., severe vomiting before onset).]

* Final Decision for Low Risk Condition: [Peptic Ulcer/Erosive/Mallory-Weiss] - [Justification.]

4. Severity Classification Decision (If Condition is peptic Ulcer):

* Consideration 1 (e.g., Severity: F1): [Features suggesting Forrest Classification type 1(F1) (e.g., active bleeding, visible vessel, active oozing).]

* Consideration 2 (e.g., Severity: F2): [Features suggesting Forrest Classification type 2(F2) (e.g., non-bleeding visible vessel, adherent clot, flat pigmented haematin on ulcer base).]

* Consideration 3 (e.g., Severity: F3): [Features suggesting Forrest Classification type 3(F3) (e.g., clean base ulcer).]

* Final Decision for Ulcer Severity: [F1/F2/F3] - [Justification.]

---

Your Final Output Format should be a valid JSON object for each patient, structured hierarchically as follows:

{

"patient_id": "{row['patient ID']}",

"risk_level": "[High-Risk / Low-Risk]",

"predicted_endoscopic_finding": "[Peptic Ulcer / Erosive Gastropathy / Esophageal Varices / Mallory–Weiss Tear / Tumor / Other]",

"forrest_classification": "F1 / F2 / F3 / Not Applicable",

"justification": "[Provide a concise clinical rationale integrating the patient's vitals, laboratory values, and clinical symptoms that justify both the selected risk level and the predicted Forrest classification grade.]"

}””
